# Characteristics and Possible Role of Bovine Sperm Head-to-Head Agglutination

**DOI:** 10.3390/cells9081865

**Published:** 2020-08-09

**Authors:** Kohei Umezu, Shouhei Kurata, Hironori Takamori, Takashi Numabe, Yuuki Hiradate, Kenshiro Hara, Kentaro Tanemura

**Affiliations:** 1 Laboratory of Animal Reproduction and Development, School of Agricultural Science, Tohoku University, Sendai, Miyagi 980-0845, Japan; shouhei.kurata.t2@dc.tohoku.ac.jp (S.K.); yuki.hiradate.d4@tohoku.ac.jp (Y.H.); kenshiro.hara.b6@tohoku.ac.jp (K.H.); 2 Miyagi Prefectural Livestock Experimental Station, Osaki, Miyagi 989-6445, Japan; takamori-hi742@pref.miyagi.lg.jp; 3 Miyagi Agricultural Development Corporation, Sendai, Miyagi 981-0914, Japan; t-numabe@miyagi-agri.com

**Keywords:** cattle, sperm agglutination, sperm mitochondria, sperm motility, sperm viability

## Abstract

Although sperm head-to-head agglutination has been reported in many mammalian species, the biological significance of this unique sperm–sperm interaction remains largely unknown. Here, we aimed to examine the functional characteristics of agglutinated bovine sperm to determine the possible role of sperm agglutination in the fertilization process. We initially examined temporal changes to the degree of head-to-head agglutination in culture, and found that bovine sperm agglutinated despite the lack of sperm agglutination inducers in medium. Sperm viability and motility were evaluated by SYBR14/PI and JC-1 staining, respectively, to identify the relationship between sperm agglutination and fertilizing ability. Agglutinated sperm had increased motility, viability, and intact mitochondrial function compared with unagglutinated sperm. Furthermore, we found that heparin significantly increased the percentage of unagglutinated sperm, but did not affect viability of both agglutinated and unagglutinated sperm, suggesting that sperm agglutination dictated the viability. In conclusion, agglutinated bovine sperm maintained viability and motility for a longer time than unagglutinated sperm. Thus, we propose that the head-to-head agglutination is a crucial sperm–sperm interaction to ensure the fertilizing ability of sperm.

## 1. Introduction

Spermatozoa are unique cells because of their mission to transmit genes from one generation to the next. After ejaculation, mammalian sperm must swim a long distance and overcome several obstacles in the female reproductive tract, and very few sperm succeed in entering the oviduct (the fertilization site) [1]. Many sperm that reach the oviduct are held in a sperm reservoir by binding to oviductal epithelial cells until the time of ovulation [2]. This attachment of sperm to oviduct cell membranes has a beneficial effect on the maintenance of sperm viability in the oviduct [3]. Generally, only a single sperm fertilizes an oocyte, despite billions of sperm ejaculated by natural mating. Considerable research has focused upon sperm competition or the difference between successful and unsuccessful sperm regarding fertilization. However, the reason why so many sperm cells exist in the female reproductive tract remains undetermined.

Spermatozoa are also recognized as heterogeneous cells. Diversity can be found in several sperm characteristics such as DNA content, epigenetic state, membrane composition, morphology, and motility [4,5]. In assisted reproductive technologies or the field of animal reproduction, selection of sperm with high quality is important for obtaining a stable and high fertilization rate during in vitro fertilization. Many researchers have investigated sperm selection using several different methods [6]. Overall, special attention has been devoted to sperm competition and selection, but not to sperm cooperation. Due to this focus, mammalian sperm are often considered to function as individual cells, despite the fact that a huge number of sperm are ejaculated at once. However, it is noteworthy that occasional physical interactions of two or more sperm such as sperm pairs, sperm rosettes, sperm rouleaux, and sperm trains have been reported in both in vitro and in vivo studies [7].

In many mammalian and non-mammalian species, it has been observed that many sperm become agglutinated with other sperm via the head, termed head-to-head agglutination [7,8]. Head-to-head agglutination of mammalian sperm may be induced by the addition of serum or egg yolk to culture medium, and also by exposing sperm to female reproductive tract fluids or to medium used for in vitro fertilization [7,9,10,11,12]. These facts suggest that sperm agglutination may play a role in the mammalian fertilization process. Indeed, it has been shown that sperm agglutination enhances sperm motility in some rodents [8,13,14]. Interestingly, in promiscuous deer mice, sperm preferentially agglutinated to sperm from the same male, not from a different male, and increased their swimming velocities as a group, suggesting sperm cooperation provides a fertilization advantage in the competitive environment of promiscuous mice [13].

In the bull, however, the biological significance of sperm agglutination remains largely unclear, despite head-to-head agglutination of bovine sperm being first reported in 1950s [15]. One study indicated that bull agglutinated sperm were more likely to maintain intact acrosomes compared with single sperm, suggesting head-to-head agglutination provides a protective role for the bovine sperm acrosome [11]. However, another study showed that the proportion of bovine sperm with intact acrosomes and plasma membranes was similar between sperm incubated in sperm-agglutinating versus non-agglutinating medium, challenging the potential protective role of sperm agglutination [12]. It is also unknown whether sperm agglutination has other roles in other sperm organelles or functions in the bull. These contradictory and limited studies on bovine sperm agglutination led us to further investigate the characteristics of agglutinated sperm to determine a possible role of sperm agglutination in the fertilization process, and to address the long-standing question of why so many sperm become agglutinated. The identification of the characteristics and function of sperm agglutination may lead to an understanding of the fertilization strategy of sperm cooperation, and provide the first description of a functional sperm group in bulls.

Thus, the objective of this study was to examine the relationship between bovine sperm agglutination and fundamental sperm characteristics such as viability and motility. To achieve this purpose, we investigated temporal changes to the rate of head-to-head agglutination, and compared sperm viability and motility rates between agglutinated and unagglutinated sperm. Additionally, we evaluated the ability of sperm to agglutinate using sperm combined from different males or different species, to determine whether bovine sperm cooperated with sperm from a single male but not multiple males, like sperm from the promiscuous deer mouse [13]. Finally, we examined the effect of heparin, which is involved in the sperm–oviduct binding process [16], upon sperm agglutination and viability to determine the molecular mechanism of sperm–sperm binding.

## 2. Materials and Methods

### 2.1. Ethics Statement

All animal care and experiments in this study were conducted in compliance with the Regulations for Animal Experiments and Related Activities at Tohoku University. The study was approved by the Tohoku University Institutional Animal Care and Use Committee (2019-003-02).

### 2.2. Sperm Preparation

Bovine sperm samples were prepared as reported previously [17]. Briefly, this study used four semen straws from different mature Japanese Black cattle bulls. The four bulls were of known fertility and each semen sample contained at least 50% progressively motile sperm after thawing. Each frozen semen sample was thawed in a water bath at 38.5 °C for 15 s and then transferred to 15 mL plastic tubes. The samples were suspended in 3 mL of bovine serum albumin-free bovine gamete medium 1 (BGM-1) [18] or IVF100 medium (Cat. No. IFP9630, Research Institute for the Functional Peptides Co., Yamagata, Japan). The albumin-free BGM-1 was designed for sperm culture and does not contain any sperm capacitation inducers nor sperm agglutination factors, while IVF100 medium was designed for in vitro fertilization and contains agglutination inducers such as bovine serum albumin and capacitation inducers such as heparin. Therefore, we defined albumin-free BGM-1 as non-capacitation medium, and IVF100 medium as a capacitation medium. After centrifugation at 430× *g* for 5 min at room temperature, the supernatant was discarded and the sediment was resuspended in non-capacitation or capacitation medium at 1 × 10^7^ cells/mL. Each sperm suspension was used for all of the experiments.

Ejaculated boar sperm was collected from three different mature boars at 1, 4, and 5 years of age, which were of known fertility. Each semen sample contained at least 50% progressively motile sperm after washing. Each sample was suspended in 2 mL of IVF100 medium, and centrifuged at 430× *g* for 5 min at room temperature. After the supernatant was discarded, the sediment was resuspended in IVF100 medium at 1 × 10^7^ cells/mL. Each boar sperm suspension was used for all experiments.

### 2.3. Sperm Agglutination Assay

Sperm suspensions derived from one bull alone or two different bulls were used in this experiment. The suspensions were incubated for 1, 2, 3, 4, 5, 24, or 48 h in non-capacitation or capacitation medium at 38.5 °C with 5% CO_2_. A small amount of each suspension was added to a glass slides, covered with glass coverslips, then observed under an inverted fluorescence microscope (BZ-X710, KEYENCE, Osaka, Japan). We obtained images from random fields (size: 1085 × 1500 μm), and counted the number of unagglutinated sperm, head-to-head agglutination of two sperm, and head-to-head agglutination of three or more sperm. Note that we counted the number of agglutinations but not the sperm number constituting an agglutination. At least 100 single sperm cells or sperm agglutinations were counted, and the percentage of each of the above three groups at each timepoint were calculated.

To examine the effect of heparin on bovine sperm agglutination, we added a final concentration of 100 μg/mL heparin sodium (Nacalai Tesque, Kyoto, Japan) to sperm suspensions before incubation or after two hours of incubation. Sperm samples were then incubated in non-capacitation medium supplemented with or without heparin for 0, 2, or 4 h. After incubation, the sperm agglutination rate was determined as above-mentioned.

### 2.4. Evaluation of Head-to-Head Agglutination of Two Sperm

To determine whether two sperm agglutinated preferentially from the same bull or randomly from different bulls, two sperm suspensions (each from a different bull) were separately stained with a 25 μM CellTrackerTM Green CMFDA Dye or 50 nM MitoTracker Red FM (Thermo Fisher Scientific) in capacitation medium for 20 min at 38.5 °C with 5% CO_2_. After staining, each suspension was centrifuged at 400× *g* for 5 min at room temperature, then the supernatant was discarded. The sediment was resuspended in the capacitation medium and centrifuged again. After the supernatant was discarded, the sediment was resuspended in the capacitation medium at 1 × 10^7^ cells/mL. The same amount of both sperm suspensions stained with different trackers was mixed together, and incubated for 1 h at 38.5 °C with 5% CO_2_, then observed under a fluorescence microscope (BZ-X710, KEYENCE). We counted the total number of two-sperm agglutinations after coincubation for 60 min, and subtracted the number of two-sperm agglutinations after the preincubation for 20 min with either CellTracker or MitoTracker from these numbers. At least 100 two-sperm agglutinations were evaluated to determine the percentage of two-sperm agglutination stained with each tracker. We also performed the same experiment using boar sperm to examine the possibility of cross-species agglutination between bovine and porcine sperm.

### 2.5. Comparison of Agglutinated and Unagglutinated Sperm Viability

Sperm viability was evaluated by SYBR14/PI staining using a commercially available kit (LIVE/DEAD Sperm Viability Kit, Thermo Fisher Scientific, Waltham, MA, USA) [19]. Briefly, sperm suspension was stained with 100 nM SYBR14 for 10 min, then with 12 μM propidium iodide (PI). After staining, the sample was incubated for 3, 5, or 24 h in the non-capacitation or capacitation medium at 38.5 °C with 5% CO_2_. Suspension samples were mounted on glass slides and covered with glass coverslips, then observed under a fluorescence microscope (BZ-X710, KEYENCE). At least 100 agglutinated sperm and 100 unagglutinated sperm were divided into live sperm (SYBR-positive and PI-negative) and dead sperm (SYBR-negative and PI-positive) to compare the viability between agglutinated sperm and unagglutinated sperm.

To examine the effect of heparin on bovine sperm viability, we added a final concentration of 100 μg/mL heparin sodium to sperm samples before incubation. The samples were then stained with SYBR/PI, and incubated in non-capacitation medium supplemented with or without heparin for 2 h. After incubation, samples were observed to calculate the PI-negative rate for each group, as above-mentioned.

### 2.6. Comparison of Motility between Agglutinated and Unagglutinated Sperm

Sperm suspension was incubated for 3, 5, or 24 h in non-capacitation medium at 38.5 °C with 5% CO_2_. After incubation, the sample was mounted on glass slides and covered with glass coverslips, and then observed using an inverted microscope (BZ-X710, KEYENCE). To calculate the percentage of motile sperm, at least 100 agglutinated sperm or 100 unagglutinated sperm were observed to identify sperm with beating flagella or no beating flagella.

### 2.7. Mitochondrial Membrane Potential of Agglutinated and Unagglutinated Sperm

To compare the quality of mitochondrial function between agglutinated and unagglutinated sperm, the mitochondrial membrane potential (MMP) of sperm was evaluated by 5,5′,6,6′-tetrachloro-1,1′,3,3′-tetraethylbenzimidazolylcarbocyanine iodide (JC-1) staining using a commercially available kit as reported previously (MitoProbe^TM^ JC-1 Assay Kit, Thermo Fisher Scientific) [20]. Briefly, sperm suspension was incubated in non-capacitation medium for 0, 5 or 24 h at 38.5 °C with 5% CO_2_. After incubation, sperm samples were incubated with 2 μM JC-1 for 30 min at 38.5 °C with 5% CO_2_ in the dark. Samples were then mounted on glass slides and covered with glass coverslips. Images were obtained under a fluorescence microscope. At a high MMP, JC-1 forms J-aggregates inside mitochondria and emits orange/red fluorescence, whereas in a low MMP state, JC-1 remains as a monomer and emits green fluorescence. At least 100 unagglutinated sperm and 100 agglutinated sperm were counted to determine the presence of sperm with high MMP and low MMP.

### 2.8. Statistical Analysis

All experiments were repeated at least three times. Data are presented as mean ± standard error (SE). Statistical analyses were carried out using the two-tailed Student’s t-test for a single comparison and the Tukey–Kramer test for multiple comparisons. Values of *p* < 0.05 were considered to indicate significant differences (* *p* < 0.05, ** *p* < 0.01). 

## 3. Results

### 3.1. Bovine Sperm Agglutinated in the Absence of Sperm Capacitation and Agglutination Inducers in Medium

**Figure 1:**
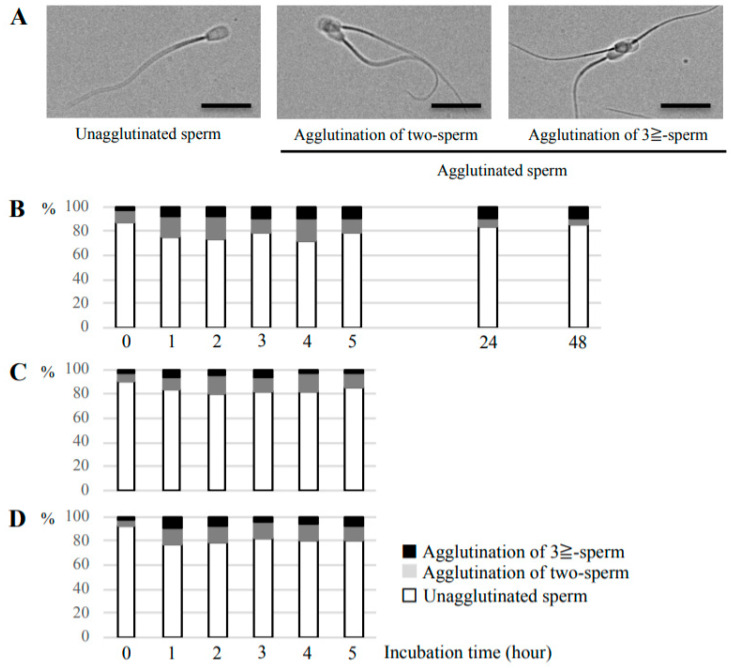
Temporal changes of head-to-head agglutination of bovine sperm. (**A**) Representative images of agglutinated and unagglutinated sperm. Left: image of single unagglutinated sperm; Middle: agglutination of two-sperm; Right: agglutination of ≥3 sperm. Bars = 20 μm. (**B**–**D**) The agglutination rate of bovine sperm was examined at 0, 1, 2, 3, 4, 5, 24, or 48 h after incubation in non-capacitation medium (**B**) or capacitation medium (**C**,**D**). The result of the sperm suspension derived from only one bull is shown in (**C**), and sperm suspension combined from two different bulls is shown in (**D**). White bar regions show the % of single sperm, gray bar regions show the % of agglutination of two sperm, and black bar regions show the % of agglutination of ≥3 sperm at each timepoint.

We initially examined the temporal change of the head-to-head agglutination rate of sperm incubated in non-capacitation medium, containing no sperm capacitation and agglutination inducers, or capacitation medium. Although most bovine sperm were not agglutinated before incubation (Video S1), the percentage of head-to-head agglutination increased to approximately 20% during the first 1 h incubation, with only minor changes during a further 4 h incubation in either medium (Figure 1). Additionally, agglutination of two sperm accounted for the major portion of head-to-head agglutination in the first 5 h of incubation. At 24 and 48 h after incubation, the agglutination rates slightly decreased in parallel with a decline in two-sperm agglutination. The agglutination rate of sperm incubated in non-capacitation medium was slightly higher than that of sperm in the capacitation medium, but there was no significant difference (Figure 1B,C). This result showed that bovine sperm agglutinated in the absence of known sperm agglutination inducers.

### 3.2. Bovine Sperm Agglutinated Randomly but Species-Specifically

**Figure 2:**
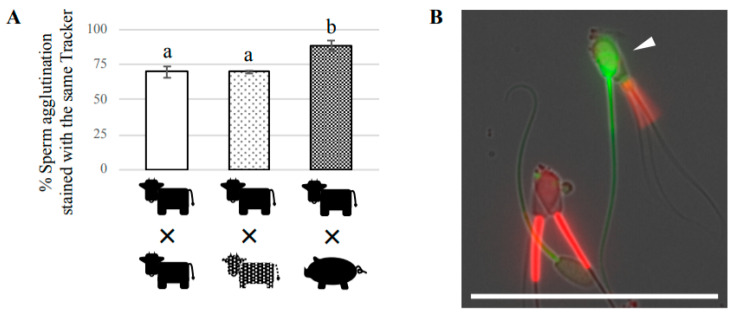
Evaluation of head-to-head agglutination of two sperm. (**A**) The percentage of two-sperm agglutination stained with the same tracker. Two sperm suspensions were separately stained with CellTracker Green or MitoTracker Red before coincubation. After washing, the same amount of sperm suspension stained with different tracker was mixed together, and co-incubated for 1 h. At least 100 two-sperm agglutinations were counted for each group. Data are shown as the mean ± SE (*n* = 3). Different letters indicate a significant difference (*p* < 0.05). (**B**) Representative superimposed image showing flagellar beating of two-sperm agglutination composed of bovine and boar sperm. Green: CellTracker (Boar sperm); Red: MitoTracker (Bovine sperm). Bar = 50 μm.

To examine whether bovine sperm preferentially agglutinate with sperm from the same bull, namely bull-specifically, or non-preferentially and randomly, we evaluated the agglutination of two sperm using CellTracker and MitoTracker. We initially confirmed that either tracker did not significantly affect the rate of sperm agglutination (Figure S1). It was also confirmed that the degree of two-sperm agglutination was similar for sperm derived from only one bull compared with sperm derived from two different bulls at all timepoints examined (Figure 1C,D). The percentage of two-sperm agglutination stained with the same tracker was 69.8% in a mixture of sperm derived from the same bull and stained with the different trackers (Figure S2). A similar result, 69.8% of two-sperm agglutination stained with the same tracker, was obtained when we used a mixture of sperm from two different bulls, each stained with a different tracker. These results suggest that bovine sperm agglutinated randomly and was not bull-specific. However, if random agglutination was present, the % of two-sperm agglutination stained with the same tracker for these two groups should be around 50%. Therefore, we counted the number of two-sperm agglutinations after preincubation for 20 min with either CellTracker or MitoTracker, and subtracted these numbers from the total number of two-sperm agglutinations after coincubation for 60 min. The resulting % of two-sperm agglutination derived from the same bull, and from mixed samples from two different bulls were 42.6% and 44.0%, respectively, supporting the idea that bovine sperm agglutinated randomly and was not bull-specific (Figure 2A).

Next, we examined the ability of bovine sperm to become agglutinated with sperm from another species. Boar sperm was selected for this experiment because boar sperm is morphologically similar to bovine sperm, and the head-to-head agglutination of boar sperm has been well-described in past studies [21,22]. We confirmed that boar sperm was agglutinated under our culture conditions, and that the levels of two-sperm agglutination in a boar sample, or in a mixture of bovine and boar sperm, were similar to those of bovine sperm (Figure S3). When bovine sperm was stained with either tracker and mixed with boar sperm stained with the other tracker, the percentage of two-sperm agglutinations stained with the same tracker was 88.7%, which was significantly higher than the % of two-sperm agglutinations for the other two groups (Figure 2A). Overall, these findings indicated that bovine sperm do not preferentially agglutinate with sperm derived from the same bull, but preferentially agglutinate with sperm from the same species. However, we also observed a few two-sperm agglutinations composed of bovine and boar sperm with beating flagella (Figure 2B), suggesting that bovine sperm have a weak ability to interact with boar sperm.

### 3.3. Agglutinated Sperm Maintained Viability and Motility for a Longer Time Compared with Unagglutinated Sperm

**Figure 3:**
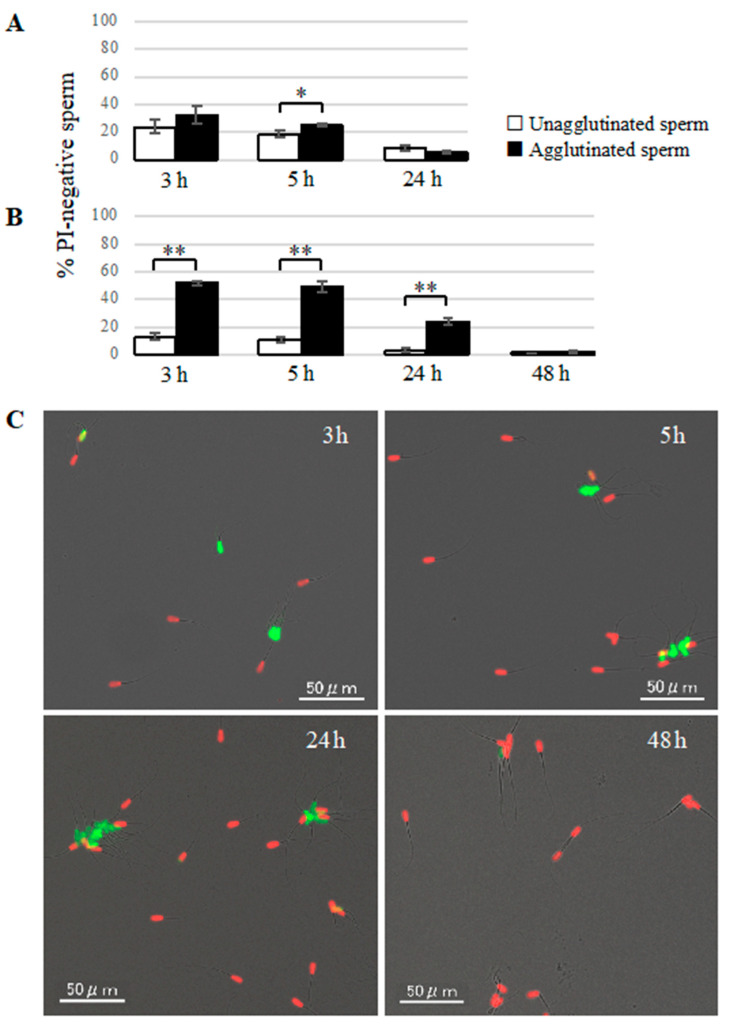
Viability of agglutinated and unagglutinated sperm. (**A**,**B**) Temporal changes in the % of PI-negative sperm in agglutinated or unagglutinated sperm. Sperm viability was evaluated by SYBR14/PI staining at 3, 5, 24, or 48 h of incubation in capacitation medium (**A**), and non-capacitation medium (**B**). White bars show the % of PI-negative cells in unagglutinated sperm, and black bars show the % of PI-negative cells in agglutinated sperm. Data shown as mean ± SE (*n* = 4; * *p* < 0.05; ** *p* < 0.01). (**C**) Representative images of sperm stained with SYBR14/PI incubated for 3, 5, 24, or 48 h in non-capacitation medium. Green: SYBR14 (live sperm); Red: PI (dead sperm). Bars = 50 μm.

We assessed the percentage of PI-negative cells in agglutinated and unagglutinated sperm incubated in capacitation or non-capacitation medium to understand the relationship between sperm viability and agglutination. The % of PI-negative agglutinated sperm was higher than that of unagglutinated sperm at 3 and 5 h incubation in either medium, but there was a more significant difference in the non-capacitation medium (Figure 3A,B). After 24 h of incubation, the % of PI-negative cells in agglutinated sperm in the non-capacitation medium was significantly higher than that of unagglutinated sperm, while there was no difference between the % of PI-negative agglutinated sperm in the capacitation medium (as more than 90% of sperm were already dead). Almost all of the sperm were dead at 48 h of incubation in the non-capacitation medium; therefore, the level of PI-negative cells were similar in agglutinated and unagglutinated sperm. Representative examples of agglutinated or unagglutinated sperm stained with SYBR14 and PI after various times of incubation in the non-capacitation medium are shown in Figure 3C. 

**Figure 4:**
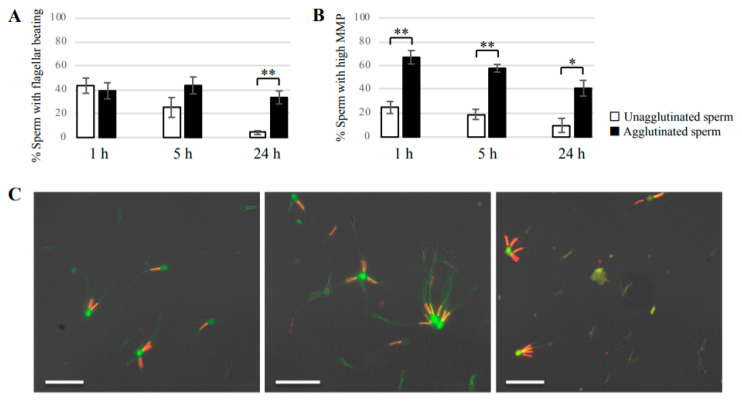
Motility and mitochondrial integrity of agglutinated and unagglutinated sperm. (**A**) The % of sperm with beating flagella in agglutinated or unagglutinated sperm. Bovine sperm were incubated for 1, 5, or 24 h in non-capacitation medium. At least 100 agglutinated sperm or 100 unagglutinated sperm were counted to determine the % of motile sperm. White bars show the % of unagglutinated sperm, and black bars show the % of agglutinated sperm. Data shown as the mean ± SE (*n* = 4; ** *p* < 0.01). (**B**) The % of sperm with a high mitochondrial membrane potential (MMP) in agglutinated or unagglutinated sperm. Sperm MMP was evaluated by JC-1 staining at 1, 5, or 24 h of incubation in non-capacitation medium. White bars show the % with high MMP in unagglutinated sperm, and black bars show that of agglutinated sperm. Data shown as the mean ± SE (*n* = 3; * *p* < 0.05; ** *p* < 0.01). (**C**) Representative images of sperm stained using the JC-1 assay. Left: image of sperm incubated for 1 h; Middle: sperm incubated for 5 h; Right: sperm incubated for 24 h. Green: sperm with a low MMP; Red or orange: sperm with a high MMP. Bars = 50 μm.

We also examined the % of agglutinated or unagglutinated sperm with beating flagella to investigate the relationship between sperm motility and agglutination. The percentage of sperm with beating flagella was similar in agglutinated and unagglutinated sperm at 1 h of incubation (Figure 4A). However, after 5 h of incubation, the % of motility for unagglutinated sperm decreased to 25.1%, compared with 43.6% of motility for agglutinated sperm (Video S2). Furthermore, 33.6% of the agglutinated sperm remained motile at 24 h of incubation, whereas most unagglutinated sperm were immotile (Video S3).

Sperm mitochondrial membrane potential (MMP) was investigated by JC-1 staining, an indicator of the energetic state of mitochondria that is related to sperm motility. The % of cells with a high MMP was significantly higher in agglutinated sperm than that of unagglutinated sperm at all timepoints examined (Figure 4B). This suggested that agglutinated sperm contained more functionally intact mitochondria compared with unagglutinated sperm. Representative examples of agglutinated or unagglutinated sperm stained with JC-1 at various times of incubation are shown in Figure 4C.

Taken together, these results indicate that agglutinated sperm had a higher level of PI-negative cells, increased motility, and increased functional mitochondria than unagglutinated sperm, suggesting that agglutinated sperm maintained viability and motility for a longer period of time in vitro.

### 3.4. Heparin Suppressed Bovine Sperm Agglutination

**Figure 5:**
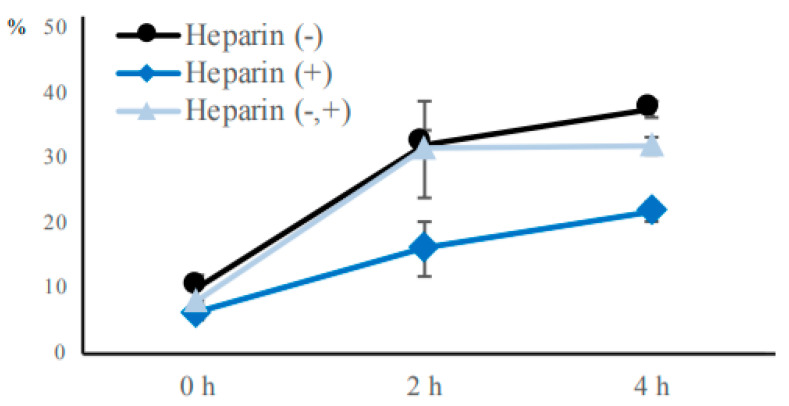
Effect of heparin on the rate of bovine sperm agglutination. The agglutination of sperm incubated without heparin (Heparin (−) group), sperm incubated with heparin (Heparin (+) group), and sperm incubated without heparin for the first 2 h and with heparin for the subsequent 2 h (Heparin (−,+) group) were examined at 0, 2, or 4 h of incubation in non-capacitation medium. The % of agglutination is shown as circles for the Heparin (−) group, triangles for the Heparin (+) group, and diamonds for the Heparin (−,+) group. Data are shown as mean ± SE (*n* = 4).

To find an inhibitor of head-to-head sperm agglutination in cattle, we focused on heparin because it specifically binds to binder of sperm (BSP) proteins on the sperm head and facilitates the detachment of sperm from the oviduct [16,23,24,25]. We hypothesized that heparin would suppress the sperm–sperm interaction, namely sperm agglutination as well as the sperm–oviduct interaction. To test this hypothesis, we compared the sperm agglutination rate of three groups: sperm incubated without heparin (Heparin (−) group), sperm incubated with heparin (Heparin (+) group), and sperm incubated without heparin for the first 2 h and with heparin for the subsequent 2 h (Heparin (−,+) group). First, we confirmed that the level of head-to-head agglutination was very low and similar for each group at the start of incubation (Figure 5). The head-to-head agglutination in the Heparin (−) group increased during the first 2 h incubation, followed by no significant change at 4 h incubation, in agreement with the findings in Figure 1. Sperm agglutination was significantly lower in the Heparin (+) group compared with the Heparin (−) group at both 2 and 4 h of incubation. This result indicates that head-to-head agglutination was suppressed by heparin. It is possible that heparin impaired the sperm from binding to each other, or alternatively, heparin facilitated the release of bound sperm. However, the level of agglutination in the Heparin (−,+) group increased to 31.5% during the first 2 h incubation without heparin, but remained constant during the subsequent 2 h incubation with heparin. This result suggests that heparin bound to BSP proteins prevented sperm head-to-head attachment. We propose that heparin acts as an inhibitor of bovine sperm head-to-head agglutination by interacting with the BSP proteins on the surface of unagglutinated sperm heads.

### 3.5. Sperm Viability Was Not Affected by Heparin

**Figure 6:**
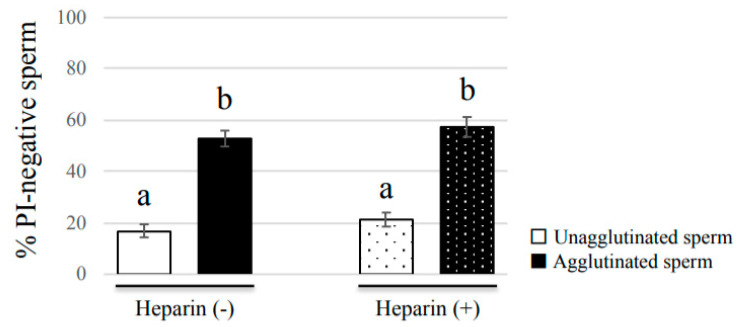
Effect of heparin on the % of PI-negative agglutinated or unagglutinated sperm. Bull sperm samples were stained with SYBR/PI, and incubated in non-capacitation medium supplemented with or without heparin for 2 h. Data shown as the mean ± SE (*n* = 4). Different letters indicate a significant difference (*p* < 0.01).

The viability assay showed that there was a close relationship between bovine sperm agglutination and sperm viability (Figure 3). However, this data may not indicate that agglutination extends the lifespan of sperm, because it may be interpreted that only live sperm, and not dead sperm, can become agglutinated. Therefore, to demonstrate that agglutination has a beneficial effect on sperm viability, we took advantage of the inhibitory effect of heparin upon sperm agglutination, and determined if sperm viability was affected by heparin. The % of PI-negative cells in both agglutinated and unagglutinated sperm were similar between the Heparin (−) and Heparin (+) groups (Figure 6). Additionally, the % of PI-negative unagglutinated sperm was significantly lower than that of agglutinated sperm, regardless of whether heparin was present or not. This result indicated that even though many of the sperm that would normally be agglutinated were unagglutinated by heparin, the viability rate of the unagglutinated sperm remained unaffected. Thus, our data suggest that sperm agglutination dictates sperm viability.

## 4. Discussion

Despite head-to-head agglutination of bovine sperm being first reported in 1950s [15], there has been little investigation of this unique sperm–sperm interaction. Here, we investigated why many bull sperm agglutinate with each other, and propose the possible role of mammalian sperm agglutination in the fertilization process.

This study found temporal changes in the degree of head-to-head agglutination of bull sperm in in vitro culture. We revealed that agglutination occurred in the capacitation medium, and also in the non-capacitation medium containing minerals, sodium lactate, and sodium pyruvate, but lacking known agglutination inducers such as serum. Although several sperm agglutination inducers have been reported in many species including cattle [7,9,10,11,12], our results indicated that bovine sperm intrinsically agglutinate for a specific purpose. To determine this purpose, we examined the relationship between sperm agglutination and fundamental functional characteristics such as sperm motility and viability.

Our study showed that agglutinated sperm exhibited increased motility, viability (more PI-negative cells), and elevated intact mitochondrial function compared with unagglutinated sperm. Furthermore, heparin inhibited sperm agglutination but did not affect the viability of unagglutinated sperm, suggesting that sperm agglutination dictated sperm viability. These findings indicate that agglutinated sperm have a longer lifespan in culture, representing a potential role for sperm head-to-head agglutination in the fertilization process. Sperm with a longer lifespan will have a greater chance to fertilize an oocyte, so sperm agglutination may enhance successful fertilization. Interestingly, sperm viability was also maintained by sperm binding to oviductal epithelial cells, the sperm–oviduct interaction [2]. Thus, sperm may be able to survive for longer periods if they physically bind to other cells. In addition to sperm viability, the sperm acrosome, an organelle located on the sperm head where it serves an important role in fertilization, was reported to be preserved by both sperm agglutination and sperm binding to the oviduct [11,26]. Furthermore, we often observed agglutinated sperm attached to the surface of glass slides (Video S4). Observations in previous in vitro studies also suggested that some sperm bound to the oviduct was agglutinated [26,27,28]. Considering these findings, we propose that these sperm–sperm and sperm–oviduct interactions are functionally similar, and may coincide in the oviduct.

It is well-known that the BSP proteins play a crucial role in attaching sperm to the oviduct in cows [16], and heparin, which specifically binds to BSP proteins on sperm, facilitates the detachment of sperm from the oviduct [23,24,25]. Our results also indicated that the head-to-head agglutination of bovine sperm was suppressed by heparin. Considering the BSP proteins are located in the sperm head [29], we propose that heparin interacts with the BSP proteins on the surface of unagglutinated sperm heads, preventing them from attaching to each other. Thus, the heparin–BSP protein interactions are involved in bovine sperm agglutination as well as sperm attachment to the oviduct. Therefore, sperm–sperm and sperm–oviduct interactions may involve similar features and regulatory molecules. Although the presence of heparin or heparin-like glycosaminoglycans has been reported in the oviduct, ooplasm, and follicular fluid [30,31,32], our results suggest that heparin was not involved in releasing sperm from agglutination. There is limited information about the process and associated factors that facilitate the detachment of sperm from each other, but it was reported that hamster sperm agglutination breaks apart when sperm motility becomes hyperactivated [7]. Interestingly, hyperactivation was also reported to be involved in releasing sperm from the oviduct [16]. Thus, we propose that sperm maintains viability by binding to other sperm or the oviduct, and are then released by hyperactivated motility or unknown factors to increase the possibility of successful fertilization and prevent polyspermy.

We found that agglutination composed of two bovine sperm accounted for the major portion of head-to-head agglutination, although we also observed agglutination ranging from two to over ten sperm. The agglutination of two sperm, termed sperm pairs, was first described in bull in this study, and has been also well-described in marsupials such as opossums [7,33]. Therefore, sperm pairs may be prevalent in mammals. By focusing on two-sperm agglutination, we also examined the ability of agglutinated bovine sperm to form pairs with sperm from a different bull, or with boar sperm. Our results showed that bovine sperm do not preferentially agglutinate with sperm from the same male, but do preferentially agglutinate with sperm from the same species. This finding contrasts with a previous study of promiscuous deer mice showing that sperm from the same male agglutinated to enhance swimming velocity, and cooperated with each other in a competitive environment [13]. It is possible that cows are not normally inseminated by several different males in a short period of time, so bovine sperm do not have selective pressure to distinguish between sperm from different males, unlike promiscuous animals. In contrast, bovine sperm are likely to have the ability to distinguish between sperm from different species. However, it should be noted that we observed some two-sperm agglutinations composed of bovine and boar sperm with beating flagella, suggesting that there may be a weak interaction between bovine and boar sperm. These findings indicate that most of the mechanisms and/or molecular factors of sperm agglutination are species-specific in mammals, although some similarity is present between bull and boar. To the best of our knowledge, this is the first description of inter-species sperm agglutination in mammals

In summary, we have demonstrated that agglutinated bovine sperm maintains viability and motility for a longer time in culture, although they do not preferentially agglutinate with sperm from the same male. We propose that head-to-head agglutination is an important sperm–sperm interaction that may enhance the fertilizing ability of sperm. Our study has revealed functional characteristics and the possible biological significance for this unique sperm–sperm interaction. This may represent the first evidence of sperm cooperation in the bull, reflecting a process also in other mammals including rodents and marsupials [7]. The extended function of agglutinated sperm may explain why so many sperm enter the female reproductive tract. Further study, notably in vivo investigations, are required to examine whether the role of sperm agglutination proposed in this study can be applied to the in vivo fertilization process in mammals.

## Supplementary Materials

The following are available online at https://www.mdpi.com/2073-4409/9/8/1865/s1, Figure S1: Head-to-head agglutination rate of the mixture of bovine and boar sperm incubated with or without CellTracker or MitoTracker. White bars show the percentage of unagglutinated sperm, gray bars show the % of two-sperm agglutination, and black bars show the % of agglutination of ≥three sperm. Figure S2: The rate of two-sperm agglutination stained with the same tracker calculated from the total number of two-sperm agglutination counted after the 60 min coincubation. Data shown as mean ± SE (*n* = 3). Different letters indicate a significant difference (*p* < 0.05). Figure S3: Temporal changes to the head-to-head agglutination rate of boar sperm. (**A**) Representative images of agglutinated and unagglutinated sperm. Left: image of unagglutinated sperm; Middle: agglutination of two sperm; Right: agglutination of ≥three sperm. (**B**) The agglutination of boar sperm was examined at 0 and 1 h after incubation in capacitation medium. White bars show the percentage of unagglutinated sperm, gray bars show the % of two-sperm agglutination, and black bars show the % of ≥three-sperm agglutination. Video S1: Movement of bovine sperm before incubation. Video S2: Flagellar movement of bovine sperm at 5 h of incubation. Video S3: Flagellar movement of bovine sperm at 24 h of incubation. Video S4: Agglutinated sperm attached to the surface of glass slides.
