# Supplementary material for: Characteristics and Possible Role of Bovine Sperm Head-to-Head Agglutination
Source: Cells. 2020 Aug 9;9(8):1865. doi: 10.3390/cells9081865 (PMC7463926; doi:10.3390/cells9081865)
Supplement: Supplementary file 1 [file cells-09-01865-s001.zip › Supplementary Materials/Figure S3_Umezu et al_2020.pdf]

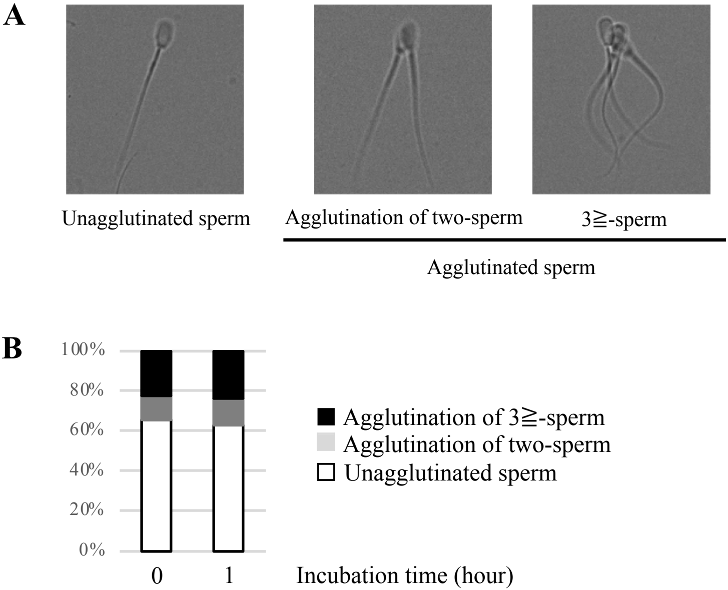
A
Unagglutinated sperm
Agglutination of two-sperm
3≧-sperm
Agglutinated sperm
B
100%
80%
60%
40%
20%
0%
0
1
Incubation time (hour)
Agglutination of 3≧-sperm
Agglutination of two-sperm
Unagglutinated sperm
