# Supplementary figures and images for: Characteristics and Possible Role of Bovine Sperm Head-to-Head Agglutination
Source: Cells. 2020 Aug 9;9(8):1865. doi: 10.3390/cells9081865 (PMC7463926; doi:10.3390/cells9081865)

% Sperm agglutination  
stained with the same Tracker

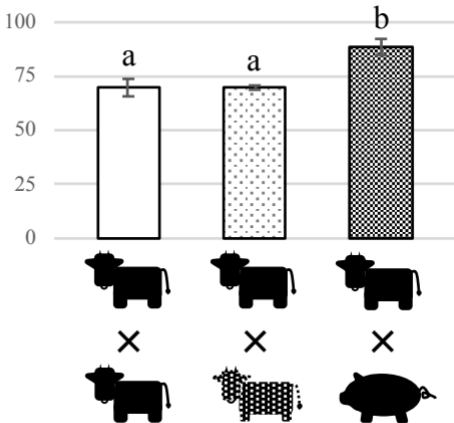

Supplement: Supplementary file 1 [file cells-09-01865-s001.zip › Supplementary Materials/Figure S2_Umezu et al_2020.pdf]

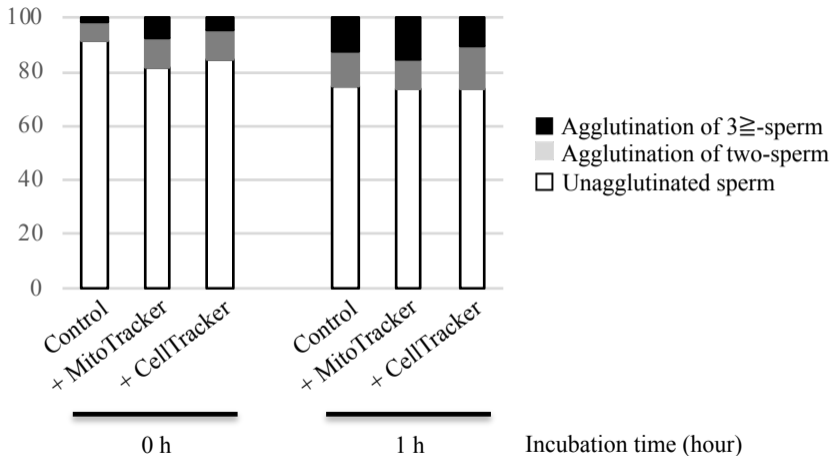

Supplement: Supplementary file 1 [file cells-09-01865-s001.zip › Supplementary Materials/Figure S1_Umezu et al_2020.pdf]
